# Betulinic Acid-Mediated Apoptosis in Human Prostate Cancer Cells Involves p53 and Nuclear Factor-Kappa B (NF-κB) Pathways

**DOI:** 10.3390/molecules22020264

**Published:** 2017-02-10

**Authors:** Eswar Shankar, Ailin Zhang, Daniel Franco, Sanjay Gupta

**Affiliations:** 1 Department of Urology, Case Western Reserve University, School of Medicine, Cleveland, OH 44106, USA; eswar.shankar@case.edu (E.S.); azhang@fhcrc.org (A.Z.); dxf176@case.edu (D.F.); 2 The Urology Institute, University Hospitals Cleveland Medical Center, Cleveland, OH 44106, USA; 3 Department of Urology, Louis Stokes Cleveland Veterans Affairs Medical Center, Cleveland, OH 44106, USA; 4 Department of Nutrition, Case Western Reserve University, Cleveland, OH 44106, USA; 5 Division of General Medical Sciences, Case Comprehensive Cancer Center, Cleveland, OH 44106, USA

**Keywords:** prostate cancer, chemoprevention, apoptosis, betulinic acid, triterpenoid

## Abstract

Defects in p53 and nuclear factor-kappa B (NF-κB) signaling pathways are frequently observed in the initiation and development of various human malignancies, including prostate cancer. Clinical studies demonstrate higher expression of NF-κB/p65/RelA, NF-κB/p50/RelB, and cRel as well as downregulation of the p53 network in primary prostate cancer specimens and in metastatic tumors. Betulinic acid (BA), is a triterpenoid that has been reported to be an effective inducer of apoptosis through modification of several signaling pathways. Our objective was to investigate the pathways involved in BA-induced apoptosis in human prostate cancer cells. We employed the androgen-responsive LNCaP cells harboring wild-type p53, and androgen-refractory DU145 cells possessing mutated p53 with high constitutive NF-κB activity. Inhibition of cell survival by BA at 10 and 20 µM concentrations occurred as a result of alteration in Bax/Bcl-2 ratio in both cell lines that led to an increased cytochrome C release, caspase activation and poly(ADP)ribose polymerase (PARP) cleavage, leading to apoptosis. BA treatment resulted in stabilization of p53 through increase in phosphorylation at Ser15 in LNCaP cells, but not in DU145 cells, and induction of cyclin kinase inhibitor p21/Waf1 in both cell types. Furthermore, treatment of both prostate cancer cells with BA decreased the phosphorylation of IκB kinase (IKK)α and I-kappa-B-alpha (IκBα) inhibiting the nuclear location of NF-κB/p65 causing cytosolic accumulation and resulting in its decreased nuclear binding. We demonstrate that BA may induce apoptosis by stabilizing p53 and downregulating NF-κB pathway in human prostate cancer cells, irrespective of the androgen association, and therefore can potentially be developed as a molecule of interest in cancer chemoprevention.

## 1. Introduction

Prostate cancer continues to be the second leading cause of death among men in the United States [1]. As per estimates from the American Cancer Society, 180,890 new cases of prostate cancer and 26,120 deaths occurred from prostate cancer in the year 2016 [2]. Prostate cancer development and progression to advance-stage disease has been linked to chronic inflammation associated with proliferative inflammatory atrophy, a putative precursor lesion [3]. The contribution by inflammation is a concerted action of cytokines and growth factors that favor tumor growth, induction of cyclooxygenase-2 in macrophages and epithelial cells, and generation of reactive oxygen and/or nitrogen species. Studies demonstrate a reasonable association between chronic inflammation and post-atrophic hyperplasia where cancer is a consequence of mutations and molecular gain of centromeric chromosome 8 [4,5]. Therefore, it is evident that the putative etiological link between chronic inflammation and prostate cancer emphasizes the need for prevention by targeting intra-prostatic inflammation [4,5].

One of the well documented pro-inflammatory response that orchestrates inflammation in the prostate is through activation of the transcription factor nuclear factor-kappa B (NF-κB) [6,7]. Deregulation of the NF-κB pathway and its aberrant stimulation has been implicated as a primordial factor in driving tumorigenesis and cancer aggressiveness that correlate with poor survival in various malignancies, including prostate cancer [8,9]. It has been well demonstrated by various researchers, including our group, that the constitutive activation of NF-κB in human prostate cancer and prostate cancer xenografts and its subsequent localization into the nucleus is considered highly predictive of disease progression/relapse and resistance to chemotherapy [8,9,10]. NF-κB activates the transcription regulatory element of the prostate-specific antigen-encoding gene, a marker of prostate cancer development and progression [11].

Additionally, the p53 tumor suppressor provides powerful intrinsic defense against cancer through its diverse function as a master regulator of cell cycle, senescence and apoptosis [12]. Globally, p53 is mutated in 50% of human cancers [13]. Although its role in prostate cancer development has been continuously debated, recent studies have shown that deregulation of p53 appears to play a significant role in the advancement and metastatic potential of the disease [14,15,16,17]. Additionally, p53 abnormalities have been observed in lymph nodes from metastatic samples of prostate cancer in patients who have not undergone hormonal therapy [18]. Evidence from the work published by Chappell et al. (2012) confirms that prostate cancer cell insensitivity to neoplastic drugs may be determined by the wild-type p53 status [19]. Therefore, the functional status of p53 is considered to be important factor in prostate cancer progression, as it dictates the overall effectiveness of the disease subjected to therapeutic intervention.

A transcriptional crosstalk exists between p53 and NF-κB in driving cancer progression. Inactivation of p53 is associated with evasion of apoptosis, whereas stimulation of NF-κB has been shown to promote resistance to programmed cell death [20,21]. p53 has been shown to be a key regulator of NF-κB repression under the influence of glucocorticoids [22]. Reports show that both p53 and NF-κB mutually repress each other’s transactivation. p53 and NF-κB have also been shown to inhibit each other’s ability to stimulate gene expression. This process is controlled by the relative levels of each transcription factor [23]. Therefore, it is speculated that suppression of p53 function may contribute to the role played by NF-κB in tumorigenesis and cancer aggressiveness. Reports also suggests that p53 and NF-κB integrate signals to drive pro-inflammatory induction in macrophages [24]. Therefore, protecting cells or tissues from inflammatory stress becomes a critical factor, and polyphenolic compounds present in plants may serve as chemopreventive agents.

Betulinic acid (BA), present in bark extracts of several species of plants, principally the white birch *Betula pubescens*, *Acmena acuminatissima* leaves, and wild jujube seeds, is the oxidation product of botulin, a lupine–derived triterpene. The biological properties of BA are well established as anti-inflammatory, anti-oxidative, anti-malarial, anti-angiogenic, anti-proliferative, and cytotoxic towards various cancer cells of human origin [25,26]. BA inhibits cancer progression and induces apoptosis in tumor cells without affecting normal cells, suggesting that it could serve as a chemopreventive agent and in combination with chemotherapy [27]. A synergistic effect in inhibiting cancer activity has been observed when BA was used in combination with Tumor necrosis factor-related apoptosis-inducing ligand (TRAIL) or ionizing radiation [28,29]. BA induces apoptosis in different cancer cells through multiple pathways, including mitochondrial pathways, p53-independent induction of p21/Waf1, upregulation of death receptors, inhibition of specificity protein (Sp) transcription factors, and interaction with other agents [30]. We have previously demonstrated that BA causes apoptosis in androgen-refractory PC-3 human prostate cancer cells, and sensitizes these cells to TNFα-induced apoptosis through suppression of NF-κB [31]. The aim of the study was to investigate the pathways involved in BA-induced apoptosis in human prostate cancer cells. Given the crosstalk between p53 and NF-κB, we hypothesized that treatment of prostate cancer cells with BA upregulates the expression of p53, thereby leading to NF-κB inactivation, and promoting apoptosis.

## 2. Results

### 2.1. Cytotoxic Effect of BA in Prostate Cancer Cells

**Figure 1:**
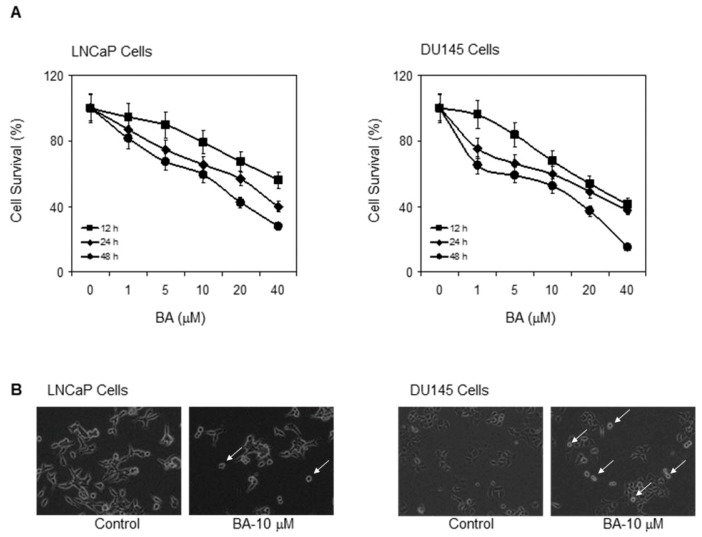
Effect of betulinic acid (BA) on human prostate cancer cell survival. (**A**) Dose- and time-dependent effect of BA in LNCaP and DU145 cells on cell survival as demonstrated by MTT assay. Representative data Mean ± SE, *n* = 8 which was repeated twice with similar results; (**B**) Microphotograph of cells treated with 10 μM BA and with vehicle only after 48 h. *Arrows*, show apoptosis characterized by marked changes in cell morphology that include contraction and membrane blebbing.

The cytotoxic effect of BA was assessed in two human prostate cancer cell lines: androgen-responsive LNCaP cells (possessing wild-type p53), and androgen-refractory DU145 cells harboring mutant p53 with higher constitutive NF-κB levels. Both cell lines were treated with 1–40 µM of BA for 12, 24 and 48 h followed by 3-[4,5-dimethylthiazol-2-yl]-2,5-diphenyl tetrazoliumbromide (MTT) assay to assess the effect on cell survival. We have previously shown that prostate cancer cells with constitutively high levels of NF-κB were more susceptible to BA treatment [31]. Here we observed that the DU145 cells showed more sensitivity to BA compared to LNCaP cells at 12 h after treatment, exhibiting loss of cell viability. After 12 h of treatment, 40 µM of BA caused 30% decreased viability in LNCaP cells, and 50%–55% in DU145 cells (Figure 1A). Treatment for 24 h and 48 h with BA in both the cell lines caused a similar shift in IC_50_ values. The 24-h treatment resulted in an IC_50_ of 38 µM, whereas the 48-h treatment yielded an IC_50_ value of 15 µM (Figure 1A,B). Cells treated with 10 µM (both LNCaP and DU145 cells) showed contraction and membrane blebbing that was typical of cells undergoing apoptosis in comparison to untreated cells (Figure 1B). Further experiments investigated whether BA has the ability to induce apoptosis in these cell lines.

**Figure 2:**
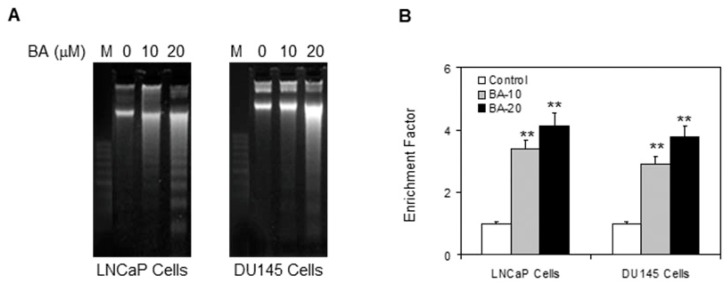
Effect of BA on DNA fragmentation and apoptosis in human prostate cancer cells. (**A**) DNA fragmentation assay. The cells were treated with vehicle or 10 or 20 μM concentration of BA for 48 h, DNA was isolated and subjected to agarose gel electrophoresis, followed by visualization of bands; (**B**) Apoptosis determined by Cell Death ELISA assay as per vendor’s protocol. Data are expressed as enrichment factor. Values represent mean ± SE of three different assays in duplicate, ** *p* < 0.001.

In the next experiment, LNCaP and DU145 cells were treated with 10 and 20 µM of BA for 48 h and apoptosis was ascertained by DNA fragmentation assay. Treatment with BA at 10 µM in both cell lines demonstrated faintly detectable fragmentation, while exposure to 20 µM of BA resulted in intensified fragmentation, confirming marked apoptosis (Figure 2A). We observed that the concentration of BA was directly correlated to the amount of DNA fragments from apoptotic cells. These results were further confirmed by ELISA assay for cell death (Figure 2B).

### 2.2. BA Induces p21/Waf1 in a p53-Dependent and Independent Manner to Cause G1 Cell Cycle Arrest in Prostate Cancer Cells

**Figure 3:**
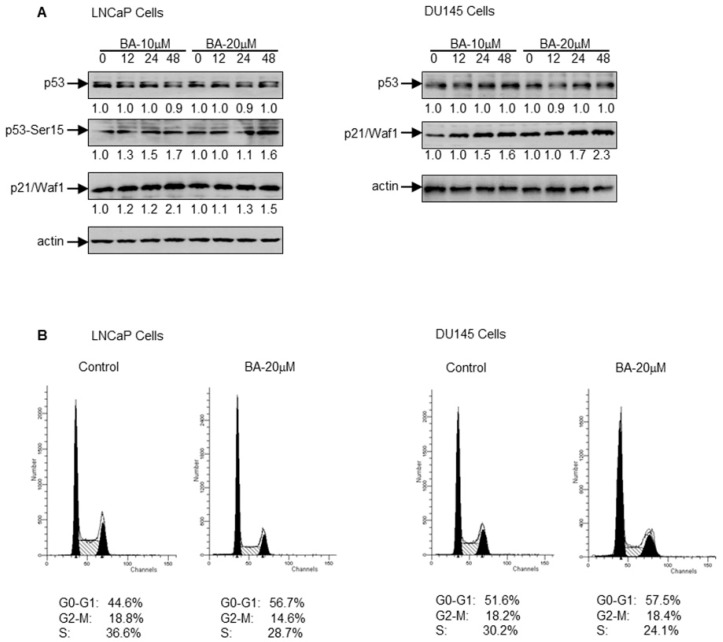
Effect of BA on p53, p21/Waf1 and cell cycle in human prostate cancer cells. (**A**) Western blot analysis for the expression of p53, Ser15–p53, p21/Waf1 in LNCaP and DU145 cells after treatment with 10 and 20 µM BA at the indicated times. The blot was stripped and reprobed with β-actin antibody to ensure equal protein loading. Numeric values represent the densitometry of protein level normalized to the loading control; (**B**) Prostate cancer cells were starved for 36 h in 1% FBS to arrest them in G1 phase of the cell cycle and exposed to 20 µM BA for 48 h, stained with PI (50 μg/mL) and analyzed by flow cytometry. Percentages of cells in sub-G1, G0–G1, S, and G2-M phase were calculated using Cell Quest and ModFit cell cycle analysis software. Data shown here are from a representative experiment repeated three times with similar results.

Next, we determined the involvement of p53 in BA-mediated cell cycle arrest and apoptosis in prostate cancer cells. In these experiments, cells were treated with 10 and 20 µM BA for 12, 24 and 48 h to determine the expression of p53, Ser15–p53 and p21/Waf1. Treatment of LNCaP cells with BA did not increase the expression of p53, but enhanced its stability through Ser15–p53 phosphorylation (Figure 3A). Treatment of DU145 cells with BA resulted in an increase in p21/Waf1 expression. Since DU145 cells possess mutant p53, no changes in Ser15–p53 phosphorylation were noted in these cells (data not shown). Cell cycle analysis revealed that BA causes G0–G1 cell cycle arrest in both cell lines; a higher percentage of G0–G1 arrest was noted in LNCaP cells (*p* < 0.001) compared to DU145 cells (*p* < 0.05) (Figure 3B). These observations suggest that cell cycle arrest in both cell lines has been driven by p21/Waf1 irrespective of p53 status.

### 2.3. BA Induces Apoptosis by Altering Bax/Bcl-2 Ratio and Causing Cyctochrome C Release in Prostate Cancer Cells

**Figure 4:**
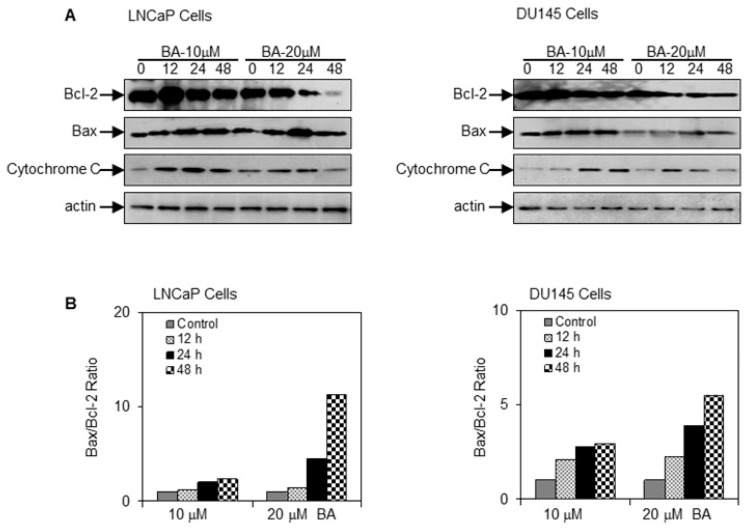
Effect of BA on Bcl-2 family member proteins and cytochrome C in human prostate cancer cells. (**A**) Immunoblotting for Bax, Bcl-2, and cytochrome C using lysates from LNCaP and DU145 cells after treatment with 10 and 20 μM concentration of BA for indicated time periods. The blot was stripped and reprobed with β-actin antibody to ensure equal protein loading. Data shown below the blots represents densitometry of the bands; (**B**) Bax/Bcl-2 ratio after densitometric analysis for proteins normalized to loading control, β-actin.

**Figure 5:**
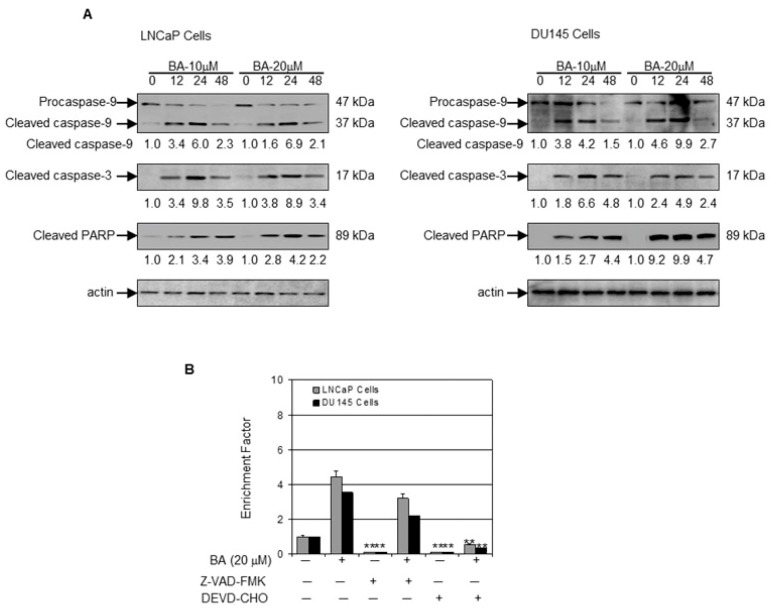
Effect of BA on markers of apoptosis in human prostate cancer cells. (**A**) Western blot analysis for caspase 9, cleaved caspase 3, and poly(ADP)ribose polymerase (PARP) expression using lysates from LNCaP and DU145 cells treated with 10 and 20 µM concentration of BA for indicated time periods. The blot was stripped and reprobed with β-actin antibody to ensure equal protein loading. Numeric values represent the densitometry of protein level normalized to the loading control; (**B**) Apoptosis in the lysates from LNCaP and DU145 cells treated with 20 µM BA in the presence and absence of pan-caspase inhibitor Z-VAD-FMK and caspase 3 inhibitor DEVD-CHO for 48 h. Data expressed as enrichment factor. Values represent mean ± SE of three different assays in duplicate, ** *p* < 0.001, compared to BA treatment.

Next, we determined the effect of BA on the expression of Bcl-2 family of proteins. Treatment with 10 and 20 µM BA for 12, 24 and 48 h of BA in LNCaP and DU145 cells showed a significant decrease in the expression of anti-apoptotic protein Bcl-2 with a concomitant increase in the expression of pro-apoptotic protein Bax. The alteration in Bax and Bcl-2 levels resulted cyctochrome C release in both the cell lines (Figure 4A,B). We further investigated the changes in caspase expression and their substrate poly(ADP)ribose polymerase (PARP) after BA exposure. Treatment of cells with 10 and 20 µM BA increased caspase 9 cleavage, followed by subsequent caspase 3 cleavage with a concomitant increase in the expression of PARP in a dose- and time-dependent manner (Figure 5A). We next investigated whether BA-mediated loss of cell viability in human prostate cancer LNCaP and DU145 cells is a result of apoptosis. To demonstrate these effects, we performed cell death detection by ELISA in the presence of pan-caspase inhibitor Z-VAD-FMK and caspase 3 inhibitor DEVD-CHO for 48 h. We observed that the pan-caspase inhibitor Z-VAD-FMK partially inhibited the apoptotic effect of BA, while caspase 3 inhibitor DEVD-CHO significantly blocked the apoptotic effect in both cell lines (Figure 5B).

### 2.4. BA-Mediated Inhibition of NF-κB Pathway in Prostate Cancer Cells

**Figure 6:**
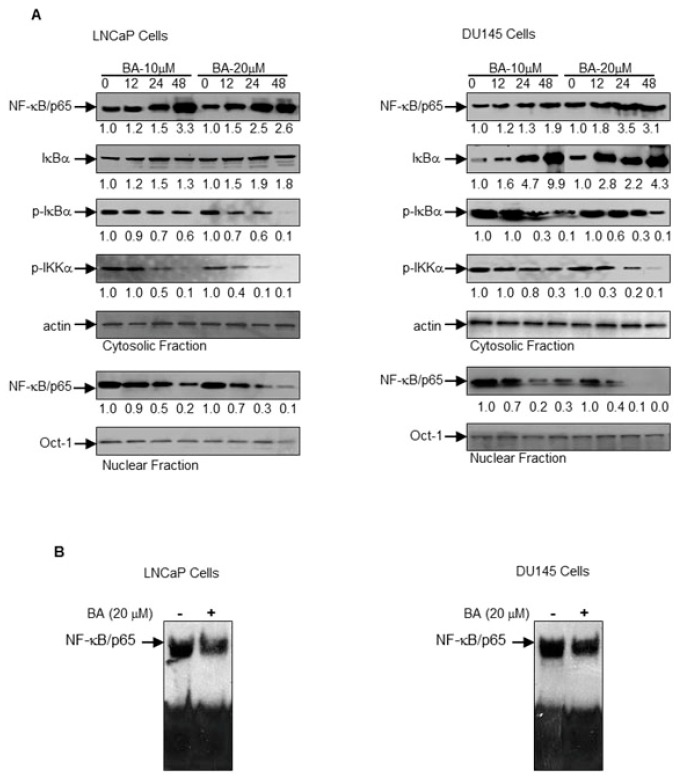
Effect of BA on nuclear factor-kappa B (NF-κB) signaling pathway in human prostate cancer cells. (**A**) Cytosolic fraction were used to determine the expression of NF-κB/p65, I-kappa-B-alpha (IκBα), p-IκBα and p-IKKα while the nuclear faction was used for NF-κB/p65 after treatment of cells with BA in dose- and time-dependent manner. The blot was stripped and reprobed with β-actin and Oct-1 antibodies to ensure equal protein loading in cytosolic and nuclear fractions. Numeric values represent the densitometry of protein level normalized to the loading control; (**B**) NF-κB/p65 DNA binding activity in LNCaP and DU145 cells treated with 20 μM BA for 24 h. Electrophoretic mobility shift assay (EMSA) was performed to determine nuclear translocation of NF-κB/p65 binding to DNA.

Next, we investigated the effect of BA on the NF-κB pathway by determining the expression of p-IκB kinase (IKK)α, I-kappa-B-alpha (IκBα), Ser32/36-IκBα and NF-κB/p65 in the LNCaP and DU145 cells. Treatment of prostate cancer cells with BA led to a decrease in the expression of p-IKKα in a time- and dose- dependent manner. IκBα phosphorylation at Ser32/36 site causes its proteasomal degradation, leading to the localization of NF-κB/p65 into the nucleus [7,8]. BA treatment decreases phosphorylation of IκBα at Ser32/36, causing increased NF-κB/p65 levels in the cytosol (Figure 6A). Further examination of NF-κB/p65 levels in the nucleus revealed decreased expression in a dose- and time- dependent manner in both cell lines (Figure 6A). Electrophoretic mobility shift assay was performed to determine the effect of BA on NF-κB/p65 nuclear binding in prostate cancer cells. BA treatment led to decreased DNA binding of NF-κB/p65 in the nucleus as a result of decreased translocation in both cell lines (Figure 6B).

## 3. Discussion

The two major signaling pathways involved in prostate cancer initiation and development include NF-κB deregulation and inactivation or loss of tumor suppressor p53 [20,21,22]. An inverse relationship exists between these proteins; mutations or defects in p53 along with constitutively active NF-κB levels in several human cancers including prostate malignancy lead to increased proliferation and suppression of apoptosis [21,22,23]. We demonstrated that BA treatment of prostate cancer cells results in induction of apoptosis, irrespective of androgen association, p53 and NF-κB status of the cells.

Wild-type p53 is considered to participate in programmed cell death in response to DNA damage in many tumor cells [12]. Under most circumstances, signals which activate the p53 response lead to rapid elevation of the p53 protein, principally through stabilization of the protein (at Ser15 and subsequently at other sites including Ser9, Ser20, Ser46 and Thr18), and activation of the DNA binding function of p53 [32]. Additionally, p53 acts as a direct transcriptional activator of downstream targets including p21/Waf1 and Bax, suggesting that p53 may activate apoptotic pathways [32,33]. Initial cell culture studies reported BA’s ability to inhibit growth of melanoma cells through induction of p53 without involvement of p21/Waf1 [34]. No accumulation of wild-type p53 protein was found upon treatment with BA in neuroblastoma cells, whereas sensitivity to BA was variably associated with loss of wild-type p53 function in p53-mutant medulloblastoma cells and in HT-29 colon carcinoma cells [35,36]. In our study, BA was shown to inhibit cell survival and induce apoptosis in LNCaP cells possessing wild-type p53 by upregulation of p21/Waf1 and induction of Bax protein. Induction of p21/Waf1 was also noted after BA exposure in p53-mutant DU145 cells causing G1 arrest, DNA damage and subsequent apoptosis. Studies have demonstrated p53-independent death mechanisms after administration of chemotherapeutic drugs or γ-irradiation and Bax-accelerated apoptosis in response to dexamethasone [37]. This data suggests that in addition to p53-restricted pathways, alternative pathways are involved in upregulation of Bax and p21/Waf1 expression and drug-induced apoptosis. Further studies are needed to understand the mechanism of BA-mediated p21/Waf1 induction in DU145 cells that harbor mutant p53.

The transcription factor NF-κB is a key mediator of the cellular stress response and inflammation, and in cells exposed to anticancer therapy NF-κB typically activates survival pathways [38]. It has been demonstrated that inhibition of NF-κB activity in cancer cell lines could reduce cell proliferation and metastatic capabilities in vivo [39]. We and others have shown that NF-κB/p65 is constitutively activated during development and progression of prostate cancer [8,9,10,11]. The major mechanism of NF-κB activation in prostate cancer cells involves aberrant activation of IKK, resulting in increased phosphorylation and instability of IκB proteins [7,8]. We have previously demonstrated that BA-induced apoptosis in androgen-refractory PC-3 human prostate cancer cells, and in addition sensitizes these cells to TNFα-induced apoptosis through suppression of NF-κB [31]. On the contrary, activation of NF-κB in response to treatment with BA has been observed in some tumor cell lines derived from neuroblastoma, glioblastoma or melanoma, demonstrating that NF-κB activation by BA may also contribute to induction of apoptosis [40]. BA activates NF-κB through increased IKK activity, phosphorylation of IκBα at serine 32/36 followed by degradation of IκBα, and nuclear translocation of the NF-κB subunit p65. On the contrary, inhibition of BA-induced NF-κB activation by different chemical inhibitors such as antioxidants, proteasome inhibitors, and IKK inhibitors also impair BA-induced apoptosis [40]. In this study, treatment of LNCaP and DU145 cells with BA caused suppression of the NF-κB pathway. We also observed that BA treatment may lead to apoptosis by decreasing the stability of IκBα and its phosphorylation at Ser32/36, thereby restricting the nuclear translocation of NF-κB/p65 and inhibiting DNA binding. Further mechanism-based studies with BA directing NF-κB signaling pathways in other human types of cancer cells are needed to validate these findings. It is tempting to speculate that combinations of BA with chemopreventive/therapeutic agents working through different pathways might prove useful in providing new treatment approaches for prostate cancer.

Numerous investigations have demonstrated that BA prevents the growth of various human cancer cells by altering key signaling pathways involved in apoptosis. BA-induced apoptosis in cancer cells has been reported to be mediated by alterations in mitochondrial function, including loss of mitochondrial permeability transition that precedes other key features of apoptosis such as activation of the caspase cascade and nuclear fragmentation [41]. A previous study by Chintharlapalli et al. (2007) reported that BA-induced apoptosis in LNCaP cells occurs by decreasing the expression of androgen receptor, leading to suppression of vascular endothelial growth factor and survivin, and caspase-dependent PARP cleavage [30]. Our data further established that treatment of LNCaP cells with BA stabilizes p53 through increase in Ser15 phosphorylation, thereby activating its downstream targets, Bax and p21/Waf1, to induce apoptosis. In DU145 cells, increase in p21/Waf1 through p53-independent mechanisms as well as decreased expression of Bcl-2 and subsequent alteration in the Bax/Bcl-2 ratio leads to increased cytochrome C release and consequent apoptosis. Our data is in agreement with other studies suggesting that BA-induced apoptosis involves depolarization of the mitochondria necessary to release cytochrome C into the cytosol, and subsequent activation of caspase 9 and caspase 3 resulting in PARP cleavage.

Crosstalk between p53 and NF-κB plays a pivotal role in cancer progression. Both these transcription factors are reported to suppress each other’s stimulation of transcription by competitively associating with coactivator p300 and CREB binding protein CBP [23]. Studies have reported that p53 represses NF-κB/p65 by a p300-dependent mechanism. Inhibition of NF-κB/p65 by p53 is shown to be achieved without altering NF-κB/p65 expression or inducible κB-DNA binding [23]. Since NF-κB pathway plays a vital role in prostate cancer cell survival, proliferation and resistance to chemotherapy, our findings suggest that this signaling pathway and activation of p53 represents key molecular targets for anticancer strategies, and that BA exemplifies a novel anticancer agent. Since BA has been selected by the National Institute of Health as a candidate for the Rapid Access to Intervention in Development program (RAID) for the development of new therapeutic agents [42], further mechanism-based studies on preclinical models of prostate cancer evaluating BA’s effect on the two major pathways are needed to validate these findings.

## 4. Material and Methods

### 4.1. Cells and Reagents

The androgen-responsive human prostate cancer LNCaP cells and the androgen-refractory DU145 cells were maintained in RPMI media purchased from HyClone Laboratories (Logan, UT, USA). Betulinic acid (≥98% purity) was obtained from A. G. Scientific, Inc. (San Diego, CA, USA). Anti-p53, anti-Ser15-p53, anti-p21/Waf1, anti-NF-κB/p65, anti-IκBα, anti-p-IκBα, anti-p-IKKα, anti-Bax, anti-Bcl-2, anti-cytochrome C and anti-PARP antibodies were purchased from Santa Cruz Biotechnology (Santa Cruz, CA, USA). Caspase 9 and Caspase 3 were purchased from Cell Signaling Technology (Danvers, MA, USA). Propidium iodide was obtained from EMD Millipore (Billerica, MA, USA). MTT (3-[4,5-dimethylthiazol-2-yl]-2,5-diphenyl tetrazoliumbromide) was purchased from Sigma-Aldrich (St. Louis, MO, USA). The Cell Death Detection ELISA^PLUS^ kit was obtained from Roche Diagnostics (Mannheim, Germany).

### 4.2. Cell Culture

LNCaP cells and the DU145 cells were maintained in RPMI 1640 containing 2.05 mM l-glutamine (Lonza, Walkersville, MD, USA) with 10% fetal bovine serum, respectively, supplemented with 1% penicillin and streptomycin in a humidified incubator at 37 °C with an atmosphere of 5% CO_2_. For experimental studies, the cells were grown to 70% confluence in monolayer and treated with indicated concentrations of BA in dimethyl sulfoxide as vehicle as previously described [31].

### 4.3. Cell Survival Assay

Briefly, cells were plated at 1 × 10^4^ cells per well in 200 μL of complete culture medium and treated with concentrations of BA prepared in dimethyl sulfoxide (DMSO) and diluted with the culture media to achieve 1–40 μM final concentrations. The concentration of DMSO remained within maximum permissible concentration of 0.1% in both control and treated samples. After incubation for 12, 24 and 48 h at 37 °C in a humidified incubator, cell viability was determined. Then, 50 μL MTT (5 mg/mL in phosphate buffered saline stock, diluted to working strength 1 mg/mL with media) was added to each well and incubated for 2 h after which the plate was centrifuged at 600× *g* for 5 min at 4 °C. The MTT solution was removed from the wells by aspiration. After careful removal of the medium, 0.1 mL of buffered DMSO was added to each well, and plates were shaken. The absorbance was recorded on a microplate reader at the wavelength of 540 nm. The effect of BA on growth inhibition was assessed as percent cell viability where vehicle-treated cells were taken as 100% viable.

### 4.4. Cell Microscopy

LNCaP and DU145 cells were plated in 100-mm dishes and left overnight in a humidified incubator at 37 °C with an atmosphere of 5% CO_2_. The following day they were treated with 10 μM of BA for 48 h and the final concentration of the vehicle dimethyl sulfoxide used to treat cells without BA was maintained at 0.1%. After 48 h treatment the cells were focused from a microscope fitted with am Olympus camera.

### 4.5. DNA Fragmentation Assay

The established hallmark of programmed cell death or apoptosis is fragmentation of chromatin to units of single or multiple nucleosomes that form the nucleosomal DNA ladder in agarose gel. The LNCaP and DU145 cells were grown to ~70% confluence and treated with 10 and 20 μM BA for 48 h. Cells were then processed for DNA isolation and fragmentation assay. The bands were visualized under a UV transilluminator, followed by digital photography as previously described [43].

### 4.6. Cell Cycle Analysis

LNCaP and DU145 cells, 70% confluent, were starved for 36 h in 1% FBS to arrest them in G1 phase of the cell cycle, after which they were treated with 20 μM BA in RPMI 1640 complete media for 48 h. Following treatment cells were collected, washed twice with chilled phosphate-buffered saline (PBS) and spun in a cold centrifuge at 600× *g* for 10 min. The pellet was fixed and resuspended in 50 μL PBS and 450 μL chilled methanol for 1 h at 4 °C. The cells were washed twice with PBS at 600× *g* for 5 min and again suspended in 500 μL PBS and incubated with 5 mL RNase (20 μg/mL final concentration) for 30 min at 37 °C. The cells were chilled over ice for 10 min and stained with propidium iodide (50 μg/mL final concentration) for 1 h. They were analyzed by Beckman Coulter XL flow cytometer (Indianapolis, IN, USA) and evaluated using Cell Quest and ModFit cell cycle analysis software (Verity Software House, Topsham, ME, USA).

### 4.7. Western Blotting

LNCaP and DU145 cells were exponentially grown and treated with 10 and 20 µM of BA for 12, 24 and 48 h. Total cell lysates were prepared using the lysis buffer (50 mM Tris-HCl, 150 mM NaCl, 1 mM EGTA, 1 mM EDTA, 20 mM NaF, 100 mM Na_3_VO_4_, 0.5% NP-40, 1% Triton X-100, 1 mM PMSF (pH 7.4)). Then, 25–30 µg protein was loaded on a 4%–12% SDS gel and transferred to a nitrocellulose membrane. In order to study the expression of proteins involved in the NF-κB pathway, cells treated with BA as indicated were subjected to subcellular fractionation to isolate the nuclear and cytosolic fraction as described previously [44]. The nuclear and cytosolic fractions were used to determine the expression of mentioned proteins involved in NF-κB pathway. Following Ponceau S visualization and blocking with 5% nonfat dry milk TBST, pH 7.4 (USB Molecular Biology Reagents, Cleveland, OH, USA) for 1 h, the membrane was probed with primary antibodies overnight at 4 °C. The following day the blots were washed and incubated with corresponding HRP-conjugated secondary antibody (Santa Cruz Biotechnology, Santa Cruz, CA, USA) and detected using Pierce ECL reagent (Fisher Scientific, Waltham, MA, USA). Bands were visualized upon autoradiography film (Denville Scientific, Metuchen, NJ, USA) exposure, quantified using digitalized scientific software program on Kodak 2000R Image Station and normalized to the loading control.

### 4.8. Apoptosis by ELISA

LNCaP and DU145 cells were treated with 20 µM BA in the presence of pan caspase inhibitors Z-VAD-FMK and the caspase 3 inhibitor, DEVD-CHO, for 48 h and ELISA for apoptosis was performed using the Cell Death Detection ELISA^PLUS^ kit following the manufacture’s protocol. Briefly, the cells after 48 h treatment with BA the cells were washed with cold PBS and lysed using Tris-HCl lysis buffer for 30 min. The lysate was clarified by centrifugation at 5000 rpm for 10 min at 4 °C and the supernatant was stored at −80 °C. Then, 50 μg of cell lysate protein were added to lysis buffer provided with the kit and pipetted on a streptavidin-coated 96-well microtiter plate to which immunoreagent mix was added and incubated for 2 h at room temperature with continuous shaking at 500 rpm. The wells were then washed with washing buffer, and color was developed by addition of substrate solution, which was read at 405 nm against the blank reference wavelength of 490 nm after 10–15 min. The enrichment factor (total amount of apoptosis) was calculated by dividing the absorbance of the sample (405 nm) by the absorbance of the controls without treatment (490 nm).

### 4.9. Electrophoretic Mobility Shift Assay

In order to study the effect of BA on the nuclear DNA binding of NF-κB/p65 electrophoretic mobility shift assay (EMSA) was performed in the LNCaP and DU145 cells. Briefly, the cells were treated with 20 µM of BA for 48 h. EMSA was performed using Lightshift™ Chemiluminiscent EMSA kit (Pierce, Rockford, IL, USA) following manufacturer’s protocol. For the Biotin 3′ end labeling of DNA in a 50 μL reaction buffer, 5 pmol of double-stranded NF-κB oligonucleotide (5′-AGTTGAGGGGACTTTCCCAGGC-3′ and 3′-TCAACTCCCCTGAAAGGGTCCG-5′) incubated in a microfuge tube with 10 μL of 5× TdT (terminal deoxynucleotidyl transferase) buffer, 5 μL of 5 μM biotin-N4-CTP, 10 U of diluted TdT, and 25 μL of ultrapure water at 37 °C for 30 min was used. A total of 2.5 μL of 0.2 M EDTA was used to stop the reaction. To extract labeled DNA, 50 μL of chloroform:isoamyl alcohol (24:1) was added to each tube and centrifuged at 13,000× *g*. The top aqueous phase containing the labeled DNA was further used for binding reactions. Each binding reaction contained 1×-binding buffer (100 mM Tris, 500 mM KCl, 10 mM dithiothretol; pH 7.5), 2.5% glycerol, 5 mM MgCl_2_, 50 ng/μL poly (dI-dC), 0.05% NP-40, 2.5 μg of nuclear extract and 20–50 fm of biotin-end-labeled target DNA. The contents were incubated at room temperature for 20 min. To this reaction mixture, 5 μL of 5× loading buffer was added, subjected to gel electrophoresis on a native polyacrylamide gel and transferred to a nylon membrane. After transfer was completed, DNA was cross-linked to the membrane at 120 mJ/cm^2^ using a UV cross-linker equipped with a 254-nm bulb. The biotin end-labeled DNA was detected using streptavidin-horseradish peroxidase conjugate and a chemiluminescent substrate. The membrane was exposed to X-ray film (XAR-5 Amersham Life Science Inc., Arlington Height, IL, USA) and developed using a Kodak film processor.

### 4.10. Statistical Analysis

One way analysis of variance (ANOVA), performed in SAS (Statistical Analysis System, Cary, NC, USA) with 95% confidence limits, was performed for determining the statistical significance. Data are presented in the figures as mean ± SE.

## 5. Conclusions

Taken together, these results suggest that BA negatively modulates prostate cancer cell growth and induces apoptosis in cell-type-specific manner which may be mediated by p21/Waf1-caused G0-G1-phase cell-cycle arrest, irrespective of the androgen association, NF-κB/p65 or p53 status of the cells.

## Abbreviations

ANOVA	analysis of variance
BA	Betulinic acid
DMSO	dimethyl sulfoxide
EMSA	Electrophoretic; mobility shift assay
IκBα	I-kappa-B-alpha
IKK	IκB kinase
NF-κB	Nuclear Factor-kappa B
PARP	poly(ADP)ribose polymerase
RAID	Rapid Access to Intervention in Development program
Sp	Specificity Protein
TRAIL	Tumor necrosis factor-related apoptosis-inducing ligand

## References

[1] Jemal A., Bray F., Center M.M., Ferlay J., Ward E., Forman D. (2011). Global cancer statistics. CA Cancer J. Clin..

[2] American Cancer Society Cancer Facts and Figures 2016. http://www.cancer.org/Cancer/ProstateCancer/index.

[3] Sfanos K.S., de Marzo A.M. (2012). Prostate cancer and inflammation the evidence. Histopathology.

[4] Stark T., Livas L., Kyprianou N. (2015). Inflammation in prostate cancer progression and therapeutic targeting. Transl. Androl. Urol..

[5] Lucia M.S., Torkko K.C. (2004). Inflammation as a target for prostate cancer chemoprevention: Pathological and laboratory rationale. J. Urol..

[6] Jin R.J., Lho Y., Connelly L., Wang Y., Yu X., Saint Jean L., Case T.C., Ellwood-Yen K., Sawyers C.L., Bhowmick N.A. (2008). The nuclear Factor-Kappa-B pathway controls the progression of prostate cancer to androgen-independent growth. Cancer Res..

[7] Nguyen D.P., Li J., Yadav S.S., Tewari A.K. (2014). Recent insights into NF-κB signaling pathways and the link between inflammation and prostate cancer. BJU Int..

[8] Shukla S., MacLennan G.T., Fu P., Patel J., Marengo S.R., Resnick M.I., Gupta S. (2004). Nuclear factor-kappaB/p65 (Rel A) is constitutively activated in human prostate adenocarcinoma and correlates with disease progression. Neoplasia.

[9] Sweeney C., Li L., Shanmugam R., Bhat-Nakshatri P., Jayaprakasan V., Baldridge L.A. (2004). Nuclear factor-kappaB is constitutively activated in prostate cancer in vitro and is overexpressed in prostatic intraepithelial neoplasia and adenocarcinoma of the prostate. Clin. Cancer Res..

[10] Shukla S., Maclennan G.T., Marengo S.R., Resnick M.I., Gupta S. (2005). Constitutive activation of PI3K-Akt and NF-kappaB during prostate cancer progression in autochthonous transgenic mouse model. Prostate.

[11] Chen C.D., Sawyers C.L. (2002). NF-kappa B activates prostate-specific antigen expression and is upregulated in androgen-independent prostate cancer. Mol. Cell. Biol..

[12] Lowe S.W., Cepero E., Evan G. (2004). Intrinsic tumour suppression. Nature.

[13] Rivlin N., Brosh R., Oren M., Rotter V. (2011). Mutations in the p53 Tumor Suppressor Gene: Important Milestones at the Various Steps of Tumorigenesis. Genes Cancer.

[14] Bookstein R., MacGrogan D., Hilsenbeck S.G., Sharkey F., Allred D.C. (1993). p53 is mutated in a subset of advanced-stage prostate cancers. Cancer Res..

[15] Heidenberg H.B., Bauer J.J., McLeod D.G., Moul J.W., Srivastava S. (1996). The role of the p53 tumor suppressor gene in prostate cancer: A possible biomarker?. Urology.

[16] Downing S.R., Russell P.J., Jackson P. (2003). Alterations of p53 are common in early stage prostate cancer. Can. J. Urol..

[17] Effert P.J., Neubauer A., Walther P.J., Liu E.T. (1992). Alterations of the P53 gene are associated with the progression of a human prostate carcinoma. J. Urol..

[18] Buhmeida A., Pyrhönen S., Laato M., Collan Y. (2006). Prognostic factors in prostate cancer. Diagn. Pathol..

[19] Chappell W.H., Lehmann B.D., Terrian D.M., Abrams S.L., Steelman L.S., McCubrey J.A. (2012). p53 expression controls prostate cancer sensitivity to chemotherapy and the MDM2 inhibitor Nutlin-3. Cell Cycle.

[20] Tergaonkar V. (2009). p53 and NF-kappaB fresh breath in the cross talk. Cell Res..

[21] Schneider G., Henrich A., Greiner G., Wolf V., Lovas A., Wieczorek M., Wagner T., Reichardt S., von Werder A., Schmid R.M. (2010). Crosstalk between stimulated NF-kappaB and the tumor suppressor p53. Oncogene.

[22] Murphy S.H., Suzuki K., Downes M., Welch G.L., de Jesus P., Miraglia L.J., Orth A.P., Chanda S.K., Evans R.M., Verma I.M. (2011). Tumor suppressor protein p53, is a regulator of NF-kappaB repression by the glucocorticoid receptor. Proc. Natl. Acad. Sci. USA.

[23] Webster G.A., Perkins N.D. (1999). Transcriptional cross talk between NF-kappaB and p53. Mol. Cell. Biol..

[24] Lowe J.M., Menendez D., Bushel P.R., Shatz M., Kirk E.L., Troester M.A., Garantziotis S., Fessler M.B., Resnick M.A. (2014). p53 and NF-κB coregulate proinflammatory gene responses in human macrophages. Cancer Res..

[25] Zhang X., Hu J., Chen Y. (2016). Betulinic acid and the pharmacological effects of tumor suppression (Review). Mol. Med. Rep..

[26] Rabi T., Gupta S. (2008). Dietary terpenoids and prostate cancer chemoprevention. Front. Biosci..

[27] Zuco V., Supino R., Righetti S.C., Cleris L., Marchesi E., Gambacorti-Passerini C., Formelli F. (2002). Selective cytotoxicity of betulinic acid on tumor cell lines, but not on normal cells. Cancer Lett..

[28] Fulda S., Jeremias I., Debatin K.M. (2004). Cooperation of betulinic acid and TRAIL to induce apoptosis in tumor cells. Oncogene.

[29] Selzer E., Pimentel E., Wacheck V., Schlegel W., Pehamberger H., Jansen B., Kodym R. (2000). Effects of betulinic acid alone and in combination with irradiation in human melanoma cells. J. Investig. Dermatol..

[30] Chintharlapalli S., Papineni S., Ramaiah S.K., Safe S. (2007). Betulinic acid inhibits prostate cancer growth through inhibition of specificity protein transcription factors. Cancer Res..

[31] Rabi T., Shukla S., Gupta S. (2008). Betulinic acid suppresses constitutive and TNFalpha-induced NF-kappaB activation and induces apoptosis in human prostate carcinoma PC-3 cells. Mol. Carcinog..

[32] Loughery J., Cox M., Smith L.M., Meek D.W. (2014). Critical role for p53-serine 15 phosphorylation in stimulating transactivation at p53-responsive promoters. Nucleic Acids Res..

[33] Riley T., Sontag E., Chen P., Levine A. (2008). Transcriptional control of human p53-regulated genes. Nat. Rev. Mol. Cell Biol..

[34] Pisha E., Chai H., Lee I.S., Chagwedera T.E., Farnsworth N.R., Cordell G.A., Beecher C.W., Fong H.H., Kinghorn A.D., Brown D.M. (1995). Discovery of betulinic acid as a selective inhibitor of human melanoma that functions by induction of apoptosis. Nat. Med..

[35] Meng R.D., El-Deiry W.S. (2001). p53-independent upregulation of KILLER/DR5 TRAIL receptor expression by glucocorticoids and interferon-gamma. Exp. Cell Res..

[36] Fulda S., Friesen C., Los M., Scaffidi C., Mier W., Benedict M., Nuñez G., Krammer P.H., Peter M.E., Debatin K.M. (1997). Betulinic acid triggers CD95 (APO-1/Fas)- and p53-independent apoptosis via activation of caspases in neuroectodermal tumors. Cancer Res..

[37] Strasser A., Harris A.W., Jacks T., Cory S. (1994). DNA damage can induce apoptosis in proliferating lymphoid cells via p53-independent mechanisms inhibitable by Bcl-2. Cell.

[38] Luo J.L., Kamata H., Karin M. (2005). IKK/NF-kappaB signaling: Balancing life and death—A new approach to cancer therapy. J. Clin. Investig..

[39] Karin M. (2006). Nuclear factor-kappaB in cancer development and progression. Nature.

[40] Takada Y., Aggarwal B.B. (2003). Betulinic acid suppresses carcinogen-induced NF-kappa B activation through inhibition of I kappa B alpha kinase and p65 phosphorylation: Abrogation of cyclooxygenase-2 and matrix metalloprotease-9. J. Immunol..

[41] Fulda S., Scaffidi C., Susin S.A., Krammer P.H., Kroemer G., Peter M.E., Debatin K.M. (1998). Activation of mitochondria and release of mitochondrial apoptogenic factors by betulinic acid. J. Biol. Chem..

[42] Udeani G.O., Zhao G.M., Geun Shin Y., Cooke B.P., Graham J., Beecher C.W., Kinghorn A.D., Pezzuto J.M. (1999). Pharmacokinetics and tissue distribution of betulinic acid in CD-1 mice. Biopharm. Drug Dispos..

[43] Srivastava J.K., Gupta S. (2007). Antiproliferative and apoptotic effects of chamomile extract in various human cancer cells. J. Agric. Food Chem..

[44] Shukla S., Gupta S. (2008). Apigenin-induced prostate cancer cell death is initiated by reactive oxygen species and p53 activation. Free Radic. Biol. Med..

